# A comparison of genomic diversity and demographic history of the North Atlantic and Southwest Atlantic southern right whales

**DOI:** 10.1111/mec.17099

**Published:** 2023-08-14

**Authors:** Carla A. Crossman, Michael C. Fontaine, Timothy R. Frasier

**Affiliations:** ^1^ Biology Department Saint Mary's University Halifax Nova Scotia Canada; ^2^ Laboratoire MIVEGEC (Université de Montpellier, CNRS 5290, IRD 224) Montpellier France; ^3^ Groningen Institute for Evolutionary Life Sciences (GELIFES) University of Groningen Groningen The Netherlands

**Keywords:** conservation genetics, demographic history, population genetics, right whales, whole genome sequencing

## Abstract

Right whales (genus *Eubalaena*) were among the first, and most extensively pursued, targets of commercial whaling. However, understanding the impacts of this persecution requires knowledge of the demographic histories of these species prior to exploitation. We used deep whole genome sequencing (~40×) of 12 North Atlantic (*E. glacialis*) and 10 Southwest Atlantic southern (*E. australis*) right whales to quantify contemporary levels of genetic diversity and infer their demographic histories over time. Using coalescent‐ and identity‐by‐descent–based modelling to estimate ancestral effective population sizes from genomic data, we demonstrate that North Atlantic right whales have lived with smaller effective population sizes (*N*_*e*_) than southern right whales in the Southwest Atlantic since their divergence and describe the decline in both populations around the time of whaling. North Atlantic right whales exhibit reduced genetic diversity and longer runs of homozygosity leading to higher inbreeding coefficients compared to the sampled population of southern right whales. This study represents the first comprehensive assessment of genome‐wide diversity of right whales in the western Atlantic and underscores the benefits of high coverage, genome‐wide datasets to help resolve long‐standing questions about how historical changes in effective population size over different time scales shape contemporary diversity estimates. This knowledge is crucial to improve our understanding of the right whales' history and inform our approaches to address contemporary conservation issues. Understanding and quantifying the cumulative impact of long‐term small *N*_*e*_, low levels of diversity and recent inbreeding on North Atlantic right whale recovery will be important next steps.

## 1 INTRODUCTION

Since its inception, conservation biology has been a field dedicated to using multidisciplinary approaches to protect biodiversity. Genetics has long served as an important tool for conservation biology through being able to detect population structure, monitor inbreeding and measure genetic diversity (Frankel, 1974; Frankham, 1995b). Advances in technology and the accessibility of genomic data have vastly increased the ways in which genomics can help inform conservation (Theissinger et al., 2023). Whole genome sequence data are easier and more accessible than ever and can help address vastly more questions with greater resolution than microsatellites, mitochondrial regions or even reduced representation sequencing (such as RADseq—Restriction‐site‐associated DNA sequencing or GBS—genotyping by sequencing) could before including understanding how population demographic history has shaped the modern genomic landscape (Allendorf et al., 2010; Ouborg et al., 2010; Taylor et al., 2021). Whole genome sequencing data can identify runs of homozygosity more accurately and precisely than reduced representation sequencing method with low marker density (Duntsch et al., 2021) and the ability to detect rare variants is again greatly improved with whole genome sequencing (North et al., 2021). These advances have greatly improved the insights we can glean from a single sample from species that are otherwise difficult to study.

Marine mammals are difficult targets for conservation: their ranges usually cross political and jurisdictional boundaries, their major threats are often related to important economic industries (e.g. fishing, shipping and coastal development) and their coastal or oceanic ranges, combined with their limited activity at the surface, make them difficult to study and monitor. Despite these difficulties, efforts across the globe are underway to help protect marine mammals and minimize anthropogenic mortalities, but often fail to consider how the historical population demography of a species may be affecting its contemporary populations.

Balaenidae is a family of baleen whales that encompasses the bowhead whale (*Balaena mysticetus*) and three species of right whales: the North Atlantic right whale (*Eubalaena glacialis*), the North Pacific right whale (*E. japonica*) and the southern right whale (*E. australis*). Female right whales become reproductively active around 8 years of age (Best et al., 2001; Hamilton et al., 1998) and are capable of reproducing every 3 years with little evidence of reproductive senescence (Best et al., 2001; Knowlton et al., 1994; Kraus et al., 2007). Right whales inhabit temperate waters around the globe migrating to slightly warmer waters in the winter to calve and back to cooler waters in the summer to feed (Braham & Rice, 1984). The high body fat content of Balaenidae (over 40%; Lockyer, 1976), combined with their extremely long baleen, made them prime target species during the whaling era.

The onset of commercial whaling by the Basques before 1100 AD (Aguilar, 1986; Reeves et al., 2007) began a multi‐century long decimation of whale populations around the globe (Reeves et al., 2007; Reeves & Smith, 2007). North Atlantic right whales in the eastern North Atlantic may have been one of the first populations of whales to be completely eradicated as the coastal nature of the species made them an easy target (Aguilar, 1986; Reeves et al., 2007). With the decimation of right whales in the eastern North Atlantic, Basque whalers then moved across to the western North Atlantic where they established whaling sites along the coasts of what are now Newfoundland, Labrador and Quebec by the mid‐1500s (e.g. Aguilar, 1986). Although it was once thought that Basque whalers caused the greatest decline in western North Atlantic right whales, reducing them from around 10,000 individuals to perhaps 1 or a few thousand (e.g. Aguilar, 1986; Cumbaa, 1986; Gaskin, 1991), more recent genetic data from bones recovered from these whaling sites show that Basques targeted bowhead whales – and not right whales – in the western North Atlantic, raising questions about the impact of Basque whaling on this population (McLeod et al., 2008, 2010; Rastogi et al., 2004). While the impact of Basque whaling is debated, most agree that the American whaling of North Atlantic right whales that followed largely ceased as they became too rare for hunting to be profitable and were considered functionally extinct (Reeves, 2001; Reeves et al., 1992, 2007). In the southern oceans, southern right whales were an equally heavily targeted species with estimates of over 140,000 individuals removed by whaling prior to the 1900s (IWC, 2001).

Today, North Atlantic right whales are listed as critically endangered by the IUCN and endangered under the U.S. Endangered Species Act (IUCN, 2022; DEPARTMENT OF COMMERCE, 2008) with an estimated 336 individuals remaining in a single population (Pettis et al., 2022). North Pacific right whales are listed as endangered by the IUCN (IUCN, 2022) and are divided into two differentiated populations on either side of the North Pacific based on mitochondrial DNA (Pastene et al., 2022). The Northeast Pacific population is made up of only ~30 individuals (Wade et al., 2011) and in the Northwest Pacific, Hakamada and Matsuoka (2016) report over 1100 individuals, although it should be noted that this estimate is based on limited sighting data and has not been reviewed by the IWC Scientific Committee. In contrast, southern right whales are listed by the IUCN as least concern with estimates of over 13,000 individuals globally (Cooke & Zerbini, 2018; International Whaling Commission, 2013). The major ocean basins in the southern hemisphere form two genetically distinct clusters of southern right whales in the South Atlantic and in the Indo‐Pacific (Patenaude et al., 2007). A number of winter nursing grounds have been described throughout their range (Baker et al., 1999; IWC, 2001), including two genetically differentiated groups in South Atlantic found off the coasts of Argentina and South Africa (Carroll et al., 2019, 2020; Patenaude et al., 2007). Southern right whale populations have experienced different recoveries post whaling with some populations experiencing near maximal growth rate (Harcourt et al., 2019), while others remain highly endangered (Cooke, 2018). The largest population in the Southwest Atlantic off the coast of Argentina was estimated to have over 4000 individuals in 2009 (International Whaling Commission, 2013) and was increasing throughout the late 2000s and early 2010s (Crespo et al., 2019).

Many of these studies assessing population structure in right whales also measured genetic diversity. Across studies, using mitochondrial DNA and microsatellites, estimates of diversity were consistently lower in North Atlantic right whales than in southern right whales from Argentina in the Southwest Atlantic (Carroll et al., 2019, 2020; Malik et al., 2000; Patenaude et al., 2007; Waldick et al., 2002). The reported levels of genetic diversity in the North Atlantic right whales are lower than the cheetah and represent one of the lowest genetic diversity levels reported for a wildlife species (Frasier et al., 2007). Interestingly, despite low overall diversity, heterozygosity in North Atlantic right whale calves is higher than expected given the genotypes of their parents, suggesting lower success of inbred foetuses versus those that are more heterozygous (Frasier et al., 2013). To date, genome‐wide assessments of diversity in right whales (both whole genome and reduced representation genome sequencing) are limited and often restricted to single individuals (e.g. Wolf et al., 2022), or do not include North Atlantic right whales (Cabrera et al., 2022).

Given the large impact whaling had on many populations, but often with incomplete records of how many individuals were actually killed, particularly during the period of Basque whaling, genetic methods to infer historical demography can be particularly informative. Different methods have been developed to estimate ancestral effective population sizes from genomic sequence data based on site frequency spectrum (SFS), and identity by descent blocks (e.g. ∂a∂i – Gutenkunst et al., 2009; MSMC – Schiffels & Durbin, 2014; IBDNe – Browning & Browning, 2015; Stairwayplot2 ‐ Liu & Fu, 2020). These models have been used to address conservation questions in marine mammals such as improving our understanding of killer whale population history and diversity (Foote et al., 2021), understanding the impact of whaling on fin whales (Wolf et al., 2022) and assessing the demographic history of the endangered vaquita (Morin et al., 2021; Robinson et al., 2022). Applying these models to right whales could improve our understanding of their history and inform our approaches for contemporary conservation. For example, if western North Atlantic right whales existed at smaller population sizes than southern right whale populations throughout much of their history, then this could help explain their different patterns of recovery from whaling.

Moreover, understanding the demographic history of a population can help explain its contemporary diversity. Populations that have undergone a bottleneck event may have been subjected to a large reduction in genetic diversity that will be evident even after the population rebounds. For example, northern elephant seals (*Mirounga angustirostrus*) underwent an extreme bottleneck event over 100 years ago where hunting reduced their numbers to only 10–20 individuals, but subsequently the species has grown to over 100,000 individuals (Hoelzel, 1999). This drastic reduction in population size is still reflected in their reduced genetic diversity compared to the southern elephant seal (*M. leonina*; Hoelzel, 1999). Unlike the rapid population decline experienced by northern elephant seals, the endangered vaquita (*Phocoena sinus*) have lived with small population sizes for hundreds of generations, yet they show little evidence of inbreeding depression and possess a low burden of deleterious mutations despite decades of low numbers (Robinson et al., 2022). Demographic history can therefore provide important context for interpreting the current landscape of genetic diversity and the potential implications for conservation.

A recent study investigated how demographic changes in baleen whales, including right whales, have been correlated with changes in abundance of their prey species and with large‐scale climatic events such as the Last Glacial Maxima (LGM; Cabrera et al., 2022). This study revealed some interesting findings related to changing ecologies of many baleen whales that could have been initiated by the LGM, focusing on the Pleistocene–Holocene transition 1 to 30 kya (Cabrera et al., 2022). The demography history results presented by Cabrera et al. (2022) are strengthened by both consistent findings across species within ocean basins and the use of genotype likelihood to account for uncertainty due to the very low coverage. However, their analyses were limited to the use of short mitochondrial markers for both Northern and southern right whales. In contrast, the nuclear dataset was limited to low‐coverage RADseq data for only a few baleen whale species and included the southern right whale, but not the North Atlantic right whale. Additionally, the confidence in the site frequency spectrum (SFS) derived from RADseq data was further limited by the use of folded SFS not exploiting the full range of the SNP frequency spectrum and by low sequencing coverage, both factors which bring important limitations to the resolution that can be achieved with such a dataset (Mona et al., 2023; Taylor et al., 2021). Therefore, the application of whole genome sequencing to the North Atlantic right whale provides a powerful opportunity to illustrate the additional conservation insights that WGS can provide. Here, we provide estimates of genomic diversity based on high‐coverage whole genome sequencing of 12 North Atlantic and 10 Southwestern Atlantic southern right whales and estimate the demographic history of each from the time of their divergence to the present. Specifically, we investigated the dynamics of the species isolation process, and their long‐term and recent changes in effective population sizes, assessing the impact of whaling on each population.

## 2 METHODS

### 2.1 DNA and library preparation

The analysed samples are part of a long‐term study on right whales that began before 1980 overseen by the North Atlantic Right Whale Consortium (NARWC). The samples used in this study were collected for other studies between 1988 and 2013, across the contemporary range of North Atlantic right whales in the Northwest Atlantic (*n* = 12) and off the coast of Argentina (*n* = 9) and South Georgia (*n* = 1) for the southern right whale samples (See Table S1 for additional collection information). Samples were collected via biopsy sampling (Brown et al., 1991; Palsbøll et al., 1991) under all required regional/federal permits. We used archived DNA from the NARWC's DNA databank housed at Saint Mary's University (Halifax, Nova Scotia) from 12 North Atlantic right whales and 10 southern right whales that had been previously extracted using standard phenol–chloroform methods (as explained in Wang et al., 2008) and stored at −20°C. A total of 1–5 μg of genomic DNA was sent to the McGill Applied Genomics Innovation Core (Montreal, Quebec, Canada) for PCR‐free library preparation using an NxSeq AmpFREE kit (Lucigen). Libraries were evenly distributed and sequenced on three lanes of an Illumina NovaSeq6000 S4 (v1.5, 2 × 150 paired ends).

### 2.2 Bioinformatic processing

Near chromosome length reference genome assemblies were downloaded for both the North Atlantic and the southern right whale from DNAZoo (www.dnazoo.org; Dudchenko et al., 2017, 2018; Table S2). We only included scaffolds longer than 1 Mbp in our analyses to avoid low‐quality assembled contigs or scaffolds and mapping biases. After variant calling, the shortest scaffold over 1 Mbp in North Atlantic Right whales was only 1.12 Mbp in length and contained only two variant sites. It was an outlier in all preliminary analyses and was therefore also excluded. The retained scaffolds included 2.17 Gbp of a total of 2.37 Gbp bases and 2.30 Gbp of 2.32 Gbp in the North Atlantic and southern right whale reference assemblies respectively. As the sister species to right whales, we chose the bowhead whale as an outgroup for our analyses. We downloaded raw reads as fastq files from the bowhead whale genome assembly from NCBI (SRR1685383; Keane et al., 2015) to be processed alongside our samples.

The bioinformatic pipelines for variant calling and variant filtration with key options and output results are provided in Figures S1 and S2 respectively. Demultiplexed fastq files were trimmed for low‐quality bases using TRIMMOMATIC v0.36 (Bolger et al., 2014). We removed residual adapters, leading bases with quality less than 20 and trimmed low‐quality bases within reads using a sliding window (window size 5 bp: mean base quality Q20). Short (<36 bp) and low‐quality reads (average base quality <30) were dropped. Paired reads were mapped against the reference genome using BWA MEM 0.7.17 (Li, 2013; Li & Durbin, 2009). In order to make the appropriate comparisons in our analyses downstream, reads from the southern right whales and the bowhead whale were mapped to both North Atlantic and southern right whale reference genomes. Bam files for each sample and lane were created and sorted with SortSam in GATK v4.1.0.0 (McKenna et al., 2010). Bam files across the three lanes for each sample were merged and duplicate reads were marked with GATK4's MergeSamFiles and MarkDuplicates tools respectively. We performed joint variant (SNPs and INDELs) calling in GATK4 using HaplotypeCaller in GVCF mode, GenomicsDBImport and GenotypeGVCFs (Figure S1). Using genomic feature repeat files available from DNAZoo, we removed variant sites from repetitive regions in the genome using BEDTOOLS INTERSECT v2.30.0 (Quinlan & Hall, 2010). We filtered variants to require individual genotype calls to have a minimum sequencing depth of 10X and genotype quality of 30 using VCFTOOLS v0.1.14 (Danecek et al., 2011). We used BCFTOOLS v1.11 (Danecek et al., 2021) to remove sites missing more than 25% of genotype calls and sites with mapping quality of less than 30 and applied a maximum depth threshold for each dataset that was calculated as two times the median depth of variant sites summed across all individuals (INFO field ‘DP’ of the VCF file) after repeat masking (NARW alone: 790X, SRW alone: 558X, all on NARW: 1370X, SRW with bowhead: 636X; Figure S2). We identified the sex chromosomes using the D‐Genies pipeline (Cabanettes & Klopp, 2018) to map our reference genomes to the well‐annotated blue whale genome assembly (GCA_009873245.3) using minimap2 (Li, 2018) and visualized the similarities with a dot‐plot. This allowed us to identify the scaffold representing the X‐chromosome and confirm that the Y‐chromosome was excluded when we only retained long scaffolds for our reference genomes. We also confirmed the identity of the X‐chromosomes by visualizing read depth across scaffolds for individuals of known sexes where read depth on the X‐chromosome was lower than on autosomal scaffolds for males. We then removed variants from the scaffold representing the X‐chromosome to include only autosomal variants in our dataset. Subsequent analyses were based on 21 scaffolds in the North Atlantic right whale genome and 20 scaffolds in the southern right whale.

### 2.3 Population genetic analyses

A flowchart of the different analyses conducted in this study with the key options is provided in Figure S3. We used KING v2.2.8 (Manichaikul et al., 2010) to identify pairwise kinship coefficients for all samples. As the presence of related individuals violates the assumptions of the analyses we used in this study, we removed from subsequent analyses three North Atlantic right whale and one southern right whale samples displaying a kinship coefficient (φ) larger than 0.177, corresponding to first‐degree relatives. Our subsequent analyses were thus performed using nine samples each from North Atlantic and southern right whales.

Variant sites in close physical proximity are often inherited together as haplotypes. Linkage disequilibrium (LD) decay can be informative for inferring historical population size variation (Nordborg & Tavaré, 2002; Park, 2012), yet analyses of population structure require unlinked SNPs (Liu et al., 2020). In order to understand the extent of LD in the right whale genomes to identify the best strategy for pruning linked sites, LD decay was calculated with PopLDdecay v3.41 (Zhang et al., 2019; Figure S4). Using PLINK v1.9 (Purcell et al., 2007), we pruned the datasets to remove linked SNPs with a mean *r* ^*2*^ threshold determined for each population (NARW: *r* ^*2*^ = .2, SRW: *r* ^*2*^ = .1). Coalescent‐based models of demographic history can also be influenced by population structure. While there is only one extant population of North Atlantic right whale, there are known subpopulations in the southern hemisphere. Most of our southern right whale samples were collected from a known, distinct population near Peninsula Valdés, Argentina, with the exception of one sample that was collected on the feeding grounds in South Georgia where whales aggregate from Argentina and less frequently South Africa (Carroll et al., 2020; Patenaude et al., 2007). To ensure the inclusion of this sample would not influence our results, we estimated population structure and individual genetic ancestry proportions using ADMIXTURE v1.3.0 (Alexander et al., 2009), varying the number of putative genetics clusters (K) from one to five, with ten replicates for each K. We plotted the cross‐validation error from our 10 independent runs to estimate the most appropriate K value and we used PONG v1.5 (Behr et al., 2016) to assess convergence across ADMIXTURE runs within our samples from each species. We also visualized population structure across species using both species mapped to North Atlantic right whales with a principal component analysis using PLINK v1.9. Finally, we calculated the level of genetic differentiation between species using Weir & Cockerham weighted *F*_*ST*_ in VFCTOOLS v0.1.14.

To assess levels of genomic diversity, we estimated observed heterozygosity, nucleotide diversity (π) and Watterson's theta (θ) in non‐overlapping 10 kb windows using SCIKIT‐ALLEL v1.3.5 (Miles et al., 2021). Genome‐wide measures were calculated as the mean of these statistics across all windows. Observed heterozygosity was calculated with both right whale populations being mapped to the North Atlantic reference assembly so that the same sites would be considered across both species. We calculate the length of all runs of homozygosity over 10 kb and estimated inbreeding coefficients by calculating the proportion of the autosomal genome covered by runs of homozygosity (ROH) of different lengths (F_ROH_) using BCFTOOLS ROH v1.16 (with the option ‐G 30). We used SCIKIT‐ALLEL v1.3.5 and VCFTOOLS v0.1.14 to repeat our estimates of F_ROH_ with different restrictions on how ROHs are identified. We used strict parameters in a multinomial HMM model implemented by SCIKIT‐ALLEL to identify ROH with the probability of observing a heterozygote in an ROH of 0 (Phet_roh = 0) due to the deep sequencing at hand delivering high confidence in our identified variants. We used default parameters in VCFTOOLS with the—LROH option and reported the maximum ROH length estimates with less than two mismatches. We plotted the length distribution of ROHs generated by BCFTOOLS to compare the timeframe of potential inbreeding events as long ROHs from recent inbreeding will be broken up by recombination over time (McQuillan et al., 2008).

The lengths of ROHs are not only a factor of time but also a factor of biological events such as inbreeding or bottleneck events that can result in long stretches of the genome being identical by descent (Kardos et al., 2017). From Thompson (2013), the time to most common recent ancestor (TMCRA) of ROHs can be estimated as:

$$\text{Time}\left(\text{generations}\right)=\frac{50}{\text{ROH}\;\text{Length}\left(\mathrm{Mb}\right)\;\text{x}\;\text{recombination}\;\text{rate}\left(\mathrm{cM}/\mathrm{Mb}\right)}$$

We estimate the age of the longest ROH tracts and the median length of ROH tracts over 10 kb for each species using a constant recombination rate of 1 cM/Mb to help understand the timing of historical inbreeding events.

### 2.4 Demographic history

We estimated the divergence dynamics of the species using MSMC‐IM (Wang et al., 2020). First, we ran MSMC2 on four North Atlantic right whale haplotypes and four southern right whale haplotypes that were mapped to the North Atlantic right whale genome and on the 16 pairwise comparisons between the two species. We combined these within‐ and between‐species outputs from MSMC2 and ran MSMC‐IM to generate cumulative migration probabilities over time. We repeated these steps to run 10 MSMC‐IM replicates using different combinations of samples/haplotypes. We also used MSMC2 v2.1.3 (Wang et al., 2020) to estimate changes in effective population size after the species diverged. We generated a list of accessible genomic regions for variant calling for each species and we removed indels and sites with missing data and phased biallelic SNPs using SHAPEIT2 (Delaneau et al., 2013). We ran 10 iterations of MSMC2 for each species, including a different combination of three samples (six haplotypes) in each run (therefore the sample from South Georgia was only included in three iterations). We plotted the results using different estimates of mutation rate to assess the robustness of our results to small changes in this parameter (Figure S8).

We generated estimates of effective population size over time based on the unfolded SFS in STAIRWAY PLOT v2.1.1 (Liu & Fu, 2020). First, we polarized the ancestral versus derived allelic states of the SNP calls for North Atlantic right whale and southern right whales setting the bowhead whale sample as the ancestral allele with a custom python script. We calculated the unfolded SFS of biallelic variant sites without missing data using SCIKIT‐ALLEL v1.3.5. We estimated the demographic history of each species using STAIRWAY PLOT v2.1.1 with a mutation rate of 0.9664 × 10^−8^ mutations/site/generation (estimate for mysticetes reported in Dornburg et al., 2012; and within the range of estimates from Suárez‐Menéndez et al., 2022) and generation time of 32 years (based on mean pre‐whaling estimates from Taylor et al., 2007).

We estimated recent demographic changes from around 4 to around 200 generations ago using IBDNe (Browning & Browning, 2015). To that aim, we first used IBDSeq (Browning & Browning, 2013) to calculate blocks of the genome that are identical by descent (IBD) using default parameters and a maximum LD coefficient between SNPs *(r* ^*2*^ max) of 1.0 to include all variants. The inclusion of all variants was based on previous studies suggesting that increasing this parameter can improve the quality of the results (Tataru et al., 2014), especially in datasets such as ours where lower values of *r* ^*2*^ max base the analyses on very few variants. We present the results for *r* ^*2*^ max = 1.0 in the main text and include a range of values in the supplementary material (Figure S9). We used filtered variant call files for each population alone, pruned to remove sites with missing data. We then used IBDNe (Browning & Browning, 2015) to generate population size estimates using a constant recombination rate of 1.0 cM/Mbp and a minimum IBD block length of 2 cM. As no recombination map is currently available, we also ran the program with a range of constant recombination rates (0.8–1.2 cM/Mbp) to reflect a range of recombination rates reported for mammals (Dumont & Payseur, 2008; Figure S9). We also estimated Tajima's D values genome wide in SCIKIT‐ALLEL as means to understand how demography is shaping diversity.

## 3 RESULTS

Nearly 7.8 billion raw reads from right whale samples were mapped to their reference genomes with a mean sequencing depth coverage of 39.48 ± 15.89 (more information on individual samples can be found in Table S1). The number of variant sites called depended on the filtering parameters required for each test and are broadly summarized in Table 1.

**TABLE 1:** Number of variant sites retained after different filtering regimes.

	Raw number of variants	After repeat masking and filtering	Only autosomes and unrelated individuals after filtering
NARW alone	5,620,253	1,249,378	1,213,686
SRW alone	21,094,633	6,389,186	6,252,485
NARW, SRW and Bowhead mapped to NARW	39,148,338	13,124,077	12,793,691
SRW with bowhead	34,368,321	11,094,247	10,841,437

*Note*: The inclusion of the bowhead whale in the same dataset allows for polarization of the SNPs and analyses of all three species when the same reference genome is required. See Figures S1 and S2 for filtering regimes used.

### 3.1 Population genetic analyses

**FIGURE 1:**
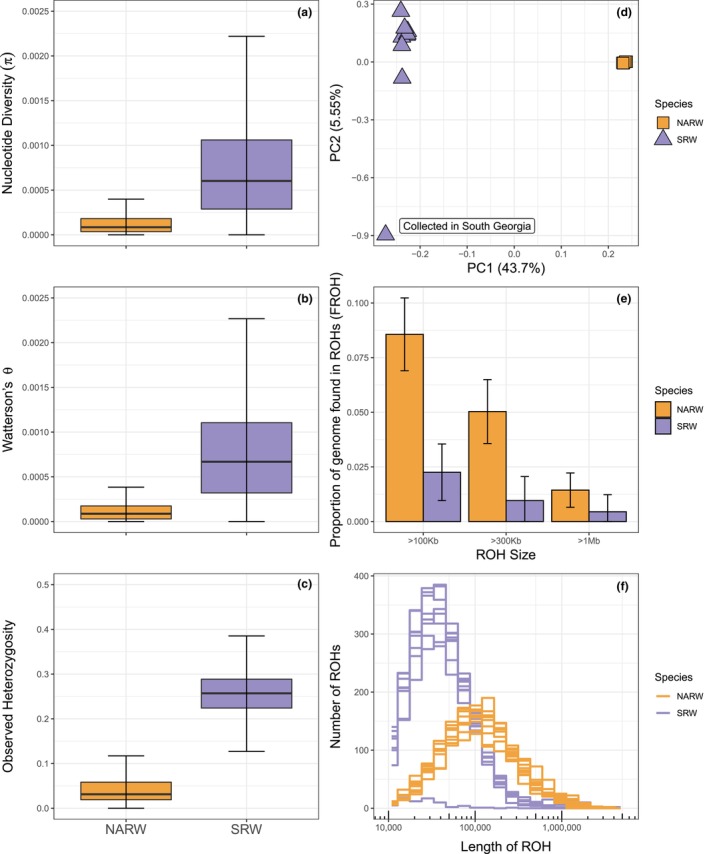
Genomic diversity of right whales. (a) Nucleotide diversity (π), (b) Watterson's θ and (c) Observed heterozygosity calculated in 10 kb non‐overlapping windows across the genome of North Atlantic right whales (left, orange) and Southern right whales (right, purple). Outliers were not plotted beyond 1.5 × IQR (interquartile range) indicated by the bars. For (c) observed heterozygosity, both species were mapped to the North Atlantic right whale genome to consider the same sites between species. (d) Principal component analysis of both right whale species: North Atlantic right whale (orange squares) and Southern right whale (purple triangle). (e) Proportion of the autosomal genome (F_ROH_) of North Atlantic right whale (orange) and southern right whale (purple) found in different ROH size classes estimated with BCFTOOLS. (f) The number of ROHs of different lengths in each species for each individual North Atlantic right whale (orange lines) and southern right whale (purple lines) estimated with BCFTOOLS.

Both right whale species showed distinct clustering on the PCA (Figure 1d; Weir & Cockerham weighted *F*_*ST*_ = 0.575). One southern right whale individual showed separation from the others along the second principal component axis, suggesting it may come from a genetically differentiated population, as may have been predicted as it was sampled from a different location (South Georgia). This subdivision was not detected in the results of ADMIXTURE which has limited power to identify a single genetically distinct sample. The cross‐validation error across 10 independent ADMIXTURE runs showed that the lowest values were obtained at *K* = 1. These results suggest that little or no population sub‐structure occurs within either set (NARW or SRW) of our samples (Figure S7). This was also suggested by the ancestry plots estimated using ADMIXTURE on both right whale species independently which displayed no convergence for K values greater than 1 (Figures S5 & S6).

When mapped to their own reference genomes, there were over five times more autosomal variant sites identified in southern right whales (6,252,485) compared to North Atlantic right whales after filtering (1,213,686). North Atlantic right whales exhibited lower genome‐wide diversity than southern right whales (mean value from non‐overlapping 10 kb windows ± SD values—NARW: nucleotide diversity π = 1.85 × 10^−4^ ± 3.57 × 10^−4^, Watterson's θ = 1.62 × 10^−4^ ± 2.57 × 10^−4^, observed heterozygosity = 0.0480 ± 0.0489; SRW: nucleotide diversity π = 8.01 × 10^−4^ ± 6.96 × 10^−4^, Watterson's θ = 8.12 × 10^−4^ ± 6.35 × 10^−4^, observed heterozygosity = 0.257 ± 0.0535; Figure 1a–c). North Atlantic right whales had a larger proportion of their autosomal genome found in ROHs than southern right whales (BCFTOOLS estimates of mean F_ROH ≥ 1Mb_ ± SD – NARW: 0.014 ± 0.0078, SRW: 0.0045 ± 0.0078; Figure 1e), indicating that North Atlantic right whales have higher inbreeding coefficients than southern right whales from the Southwest Atlantic. These results were relatively consistent across different methods (Table S3). The length of ROHs also differed between populations with our southern right whales having shorter ROHs compared to the greater abundance of long ROHs found in North Atlantic right whales (Figure 1f).

Using the median value of ROH >10 kb (NARW: 105,976 bp, SRW: 36,883 bp), we estimate that most ROHs were formed in North Atlantic right whales ~ 472 generations ago (~15,100 years ago based on a generation time of 32 years as estimated by Taylor et al. (2007)) and 1355 generations (~43,360 years) ago in Southwest Atlantic southern right whales. The longest ROHs in both species were ~ 2–4 Mb long and were likely formed 12.5–25 generations (400–800 years) ago.

### 3.2 Demographic history

**FIGURE 2:**
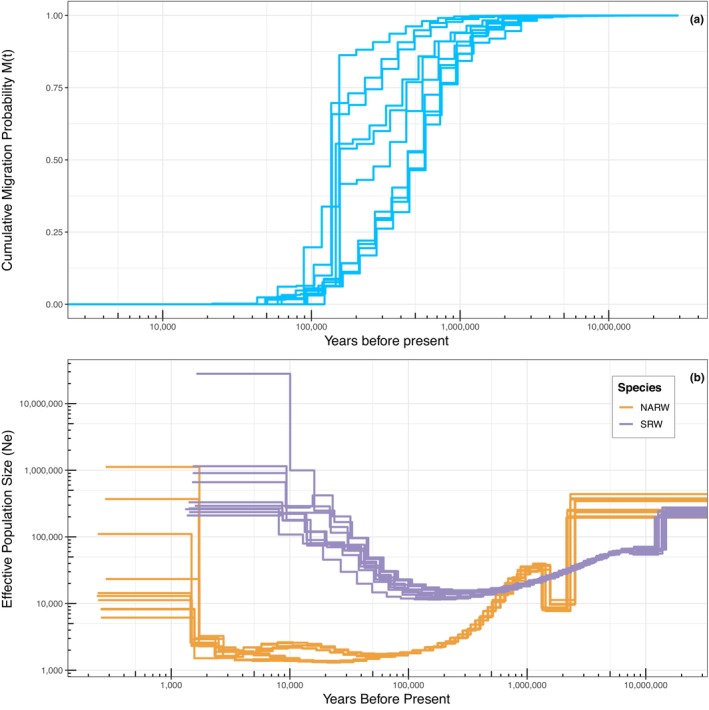
(a) Ten iterations of MSMC‐IM estimating the cumulative migration probability M(t) between North Atlantic and southern right whales over time. Estimated divergence time identified by the shaded area as M(t) dropping below 0.5 as suggested by Wang et al. (2020) and Schiffels and Wang (2020). (b) Ten estimates of effective population size through time for each North Atlantic (orange) and southern right whale (purple) generated with MSMC2.

**FIGURE 3:**
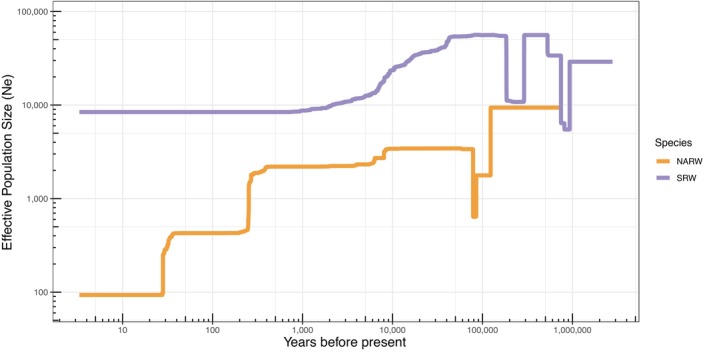
Estimates of effective population size of North Atlantic (orange) and southern right whales (purple) through time based on the site frequency spectrum in STAIRWAY PLOT2. The shaded regions represent the 2.5%–97.5% confidence limits for 200 estimates.

The cumulative migration probabilities (M(t)) between North Atlantic and southern right whales estimated with MSMC‐IM identified the putative split between the two species as occurring ~ between 124 and 480 kya, when the M(t) dropped below 0.5 (Figure 2a). Visually, we confirmed this putative split time with the effective population size (N_e_) estimates from STAIRWAY PLOT2 converging around 200–300 kya (Figure 3).

Following divergence, MSMC2 reported a decrease in *N*_*e*_ in North Atlantic right whales that plateaued around 2000 and remained relatively constant until this method lost resolution a few thousand years ago (Figure 2b). STAIRWAY PLOT2 identified a similar pattern of post‐divergence decline, followed by a plateau of *N*_*e*_ values c. 2000 between 100 and 500 ya (Figure 3). The initial post‐divergence decline identified by these methods could also be indicative of emerging population structure between the eastern and western North Atlantic. In the most recent times (<500 ya), *N*_*e*_ kept decreasing in two steps: a first one occurring between 500 ya and 200 ya with a *N*_*e*_ contraction from ~ 2000 to 500, and a second one, though associated with more uncertainty as shown by the large confidence intervals, indicating an additional fivefold contraction ~50 ya. In southern right whales, after the species diverged both methods estimate that effective population size remained much higher than in the North Atlantic right whale. MSMC2 estimated a slight increase in effective population size between 100,000 and 10,000 years ago, whereas STAIRWAY PLOT2 identified a steadier decline in the population in that timeframe (Figures 2b and 3). The broad patterns in these results were found across a range of mutation rates used in MSMC2 (Figure S8).

**FIGURE 4:**
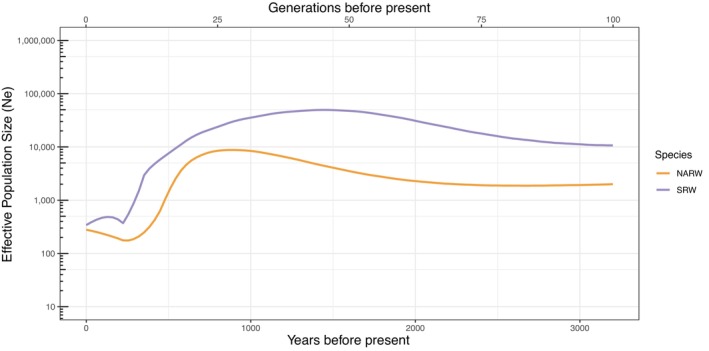
Effective population size in recent history as estimated by IBDNe for North Atlantic (orange) and southern right whale (purple) using a constant recombination rate of 1.0 cm/Mb and a generation time of 32 years. 95% confidence intervals are depicted by the shaded areas.

The most contemporary estimates of effective population size were best captured by IBDNe (Figure 4). From 3kya to roughly 1kya, IBDNe identified relatively stable effective population size in North Atlantic right whales of approximately 1000 and Southwest Atlantic southern right whales at >10,000. Within the last 1000 years, a steep decline was detected in both species lasting until ~ 225 years ago, when the effective population size of North Atlantic right whales was below 200 and southern right whales was around 350. Both populations began to show growth in *N*_*e*_ following this bottleneck event. A slight decline was detected within the last 100 years (three generations) in Southwest Atlantic southern right whales; however, this should be interpreted with caution as the methods used have much lower resolution in this recent timeframe (Browning & Browning, 2015). These general patterns with a decline in *N*_*e*_ until approximately 225 years ago, followed by increasing *N*_*e*_ in more recent years were consistent across different *r* ^*2*^ max values and recombination rates (Figure S9). The mean Tajima's D value was slightly positive in North Atlantic right whales (0.298 ± 1.209) and slightly negative in our southern right whales (−0.150 ± 0.816), potentially reflecting these differences in their demographic histories.

## 4 DISCUSSION

Using whole genome sequence data from nine unrelated individuals from both North Atlantic and southern right whales from the Southwest Atlantic, we found lower levels of genetic diversity and higher inbreeding coefficients in North Atlantic right whales compared to southern right whales. We also described the changes in effective population size over time in each of these species since their divergence. We estimate a divergence time of ~ 124–480 kya, which is more recent than has been described in other studies (McGowen et al., 2009: 95% HPD 0.34–1.28 MYA; McGowen et al., 2020: six‐partition AR model 95% CI 3.65–5.11 MYA, six‐partition IR model 95% CI 1.67–2.11 MYA; Slater et al., 2017: 95% HPD 0.45–1.43 MYA). This slightly more recent split estimate is most likely related to the population genetic analytical framework we have used in this study using MSMC‐IM and to the different mutation rates used here compared to McGowen et al. (2020). Our analytical approach contrasts with more ‘classic’ phylogenetic approaches by modelling patterns of lineage sorting under the cross coalescent rate (CCR) among species from which the rate of cessation of gene flow can be estimated. The recent split time estimated by the CCR decay of MSMC‐IM could reflect potential secondary contact that may have occurred between North and southern Atlantic right whales during the Quaternary glaciations. Furthermore, while our study used pedigree‐based mutation rate estimates, the phylogenetic approaches of McGowen et al. (2020) used a relaxed molecular clock calibrated with fossil records to estimate divergence times. While both approaches are valid in their respective statistical framework, they usually lead to one‐ or two‐order‐magnitude differences in split time estimates (Henn et al., 2008; Ho, Shapiro, et al., 2007; Howell et al., 2003). This discrepancy between the CCR decay approach implemented in MSMC‐IM and more traditional phylogenetic approaches comes from the fact that phylogenetic approaches as used in McGowen et al. (2020) rely on estimating substitution rate (complete nucleotide replacement), ignoring within‐species polymorphic variation and the inter‐loci gene‐tree discordance due to incomplete lineage sorting and gene flow. Recently developed phylogenetic approaches based on the multi‐species coalescent now allow incorporating these properties to better estimate species diversification processes (Flouri et al., 2018; Jiao et al., 2020, 2021; Müller et al., 2017, 2018; Rannala & Yang, 2017; Solís‐Lemus et al., 2016). While not the primary focus of this study, understanding the timing and rate associated with how the species diverged with dedicated approaches warrants further investigation specific to the *Eubalaena* and could benefit from the inclusion of additional samples through the range of the southern right whale.

After diverging, southern right whales had effective population sizes nearly 10 times greater than North Atlantic right whales for thousands of years. We also detected a rapid and recent decline in both populations over the past thousand years, as expected based on the history of whaling (Aguilar, 1986; Rastogi et al., 2004; Reeves et al., 1999). The recent decline in Southwest Atlantic southern right whales was only detected by the most sensitive analysis of IBDNe relying on IBD tract lengths and was not detected based on site frequency spectrum data used by STAIRWAY PLOT2. This analysis suggests the bottleneck occurred more recently than in the North Atlantic, as predicted by the onset of industrial whaling beginning a couple of centuries later into the 1700s (International Whaling Commission, 2013).

The history of whaling over the past thousand years is clearly imprinted in the genomic diversity of both North Atlantic and southern right whales. The longest ROHs identified for both species were likely formed 400–800 years ago as effective population sizes drastically declined (see discussion below on how parameter selection can lead to a small discrepancy with the timing of historical events). While most European and American whaling ended in the mid‐1900s (Reeves et al., 2007; Reeves & Smith, 2007), illegal Soviet whaling continued in the Southern Oceans and over 3300 southern right whales were estimated to have been killed between 1951 and 1971 (Tormosov et al., 1998). This spike in whaling efforts might explain a small dip in the effective population size of southern right whales from the Southwest Atlantic in the past few generations. However, some caution is warranted with this interpretation as the power to detect changes in effective population size during this timeframe is low (Browning & Browning, 2015).

Our results showed slightly different patterns of effective population size over time generated by MSMC2 and STAIRWAY PLOT2 between 10 and 100 KYA in southern right whales. Discrepancies produced by these methods could be explained by gene flow from other parts of their range and/or could provide some evidence of selection (Boitard et al., 2022; Johri et al., 2021). Historical population size estimates can be biased by population structure as it violates the panmictic assumptions of these models (Heller et al., 2013; Ho & Shapiro, 2011). In our population structure analysis, one southern right whale sample was slightly differentiated from the others and was collected from a different location (on the feeding grounds of South Georgia) where individuals from different breeding populations aggregate (Carroll et al., 2020; Patenaude et al., 2007). Therefore, we repeated our analyses of IBDNe and STAIRWAY PLOT2 excluding this individual (Figures S10 and S11) and confirmed that its inclusion had little impact on our inferences of demographic history. The timing of the discrepancy between MSMC2 and STAIRWAY PLOT2 also corresponds with the age of median ROH length in southern right whales (~43,360 years ago) adding additional evidence for interesting dynamics during this timeframe. The extent of selection, both historical and contemporary in southern right whales from the Southwest Atlantic, remains unknown.

North Atlantic right whales have been extirpated from the Northeast Atlantic and only a single extant population remains in the Northwest Atlantic. Estimates of historical effective population size of the North Atlantic right whale population could therefore be inflated due to past gene flow with the unsampled population from the Northeast Atlantic – or in contrast, underestimate past effective population size of the species as a whole. Likewise, gene flow from an unsampled population of southern right whales (Carroll et al., 2019, 2020) could inflate our estimates of diversity and effective population size and therefore a precautionary approach should be taken in using these estimates for conservation planning. The potential for current and future gene flow, the relatively short nature of the bottleneck event, recent increasing population sizes (Crespo et al., 2019), the lower inbreeding coefficient and the excess of rare variants in the Southwest Atlantic right whale population are all promising signs for the resiliency and recovery of this population.

**FIGURE 5:**
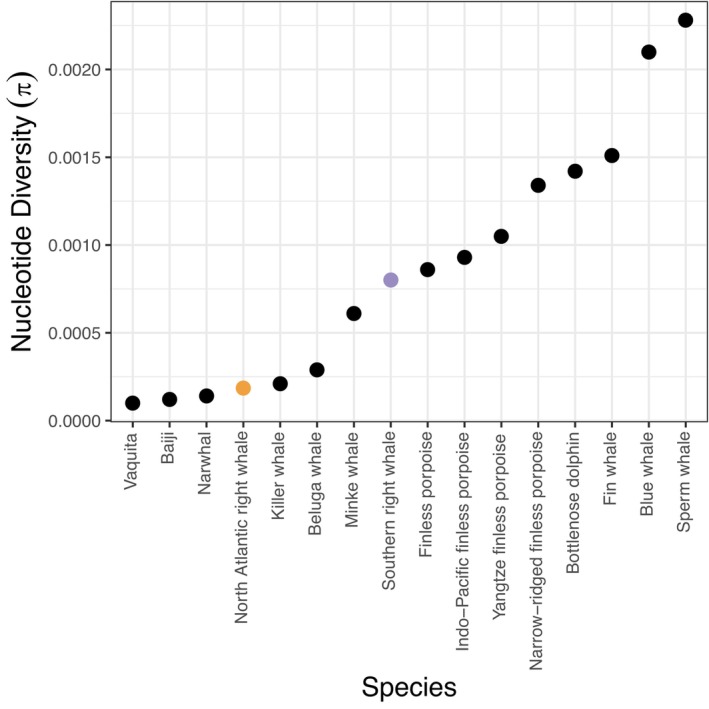
Nucleotide diversity reported for other cetacean species based on genomic datasets. Raw data and sources for the non‐right whale species can be found in Table S4.

Small population sizes have a number of consequences: (1) low population sizes can erode genetic diversity (Allendorf, 1986; Wright, 1931), (2) reduced mating opportunities increase the inbreeding coefficient as average pairwise relatedness between individuals increases (Charlesworth & Charlesworth, 1987; Charlesworth & Willis, 2009; Wright, 1922) and (3) long‐term small population size can lead to long tracks of homozygosity, exposing deleterious alleles and thus reducing the strength of purifying selection thereby altering the genetic load in a population (Frankham, 1995a, 1999; Grossen et al., 2020; Keller & Waller, 2002). North Atlantic right whales possess extremely low genomic diversity—lower than the cheetah (Dobrynin et al., 2015), on par with the Iberian lynx (Westbury et al., 2018) and one of the lowest nucleotide diversity values recorded for cetaceans (Morin et al., 2021; Figure 5; Table S4). We also see evidence of both long‐term and recent inbreeding through the high proportion of the genome found in ROHs (F_ROH_) and a higher proportion of long ROHs indicative of recent inbreeding events where not enough time has passed to allow recombination to break them up. The extent of historical and/or contemporary purifying or balancing selection in the North Atlantic right whale is unknown and remains an important question for future study. Therefore, the cumulative effects of long‐term small effective population size of North Atlantic right whales are yet to be determined.

The estimates of demographic history are based on coalescent models using site frequency spectrum or runs of homozygosity and are influenced not only by the data themselves but also by factors such as generation time, mutation rate, recombination rate and selection. An inherent assumption of many biological models is non‐overlapping generations, which is violated for most mammalian species. In right whales, a female can be reproductively active for decades (Hamilton et al., 1998) and therefore be calving at the same time as her granddaughters. This assumption is largely unavoidable with long‐lived mammalian studies but should be recognized as a potential source of bias where *N*_*e*_ is likely underestimated and bottlenecks may be detected earlier than they occurred (Charbonnel et al., 2022; Waples et al., 2014). Mutation rate is used in estimating changes in effective population sizes over time. Estimates of mutation rate based on phylogenies have been generated for many species including baleen whales (Dornburg et al., 2012; Jackson et al., 2009), but these do not account for within‐species variation and therefore likely underestimate the true mutation rate. Pedigree‐based estimates of mutation rate, or those based on ancient DNA, should therefore be more accurate (Ho et al., 2008; Ho, Kolokotronis, & Allaby, 2007), but these are still lacking for many species. The mutation rate used in this study is similar to those reported for other mammals based on pedigree estimates, including closely related whale species (Suárez‐Menéndez et al., 2022), and the patterns in our results are fairly robust to small changes in mutation rate (Figure S8). Recombination rate will help dictate the break‐up of IBD blocks over time. In this study, we assumed a constant recombination rate across the genome, which is inherently incorrect. Our results, however, are quite robust to a range of recombination rates expected for mammals (Figure S9), and therefore, while a variable recombination rate map would be more accurate, no recombination maps for right whales or related species were available and we do not feel it would alter the broad patterns found in our results. Finally, accounting for potential effects of selection is difficult, and while we may have some evidence of historical selection in southern right whales as indicated by a discrepancy of population trends from different methods, the patterns in our results, especially in more recent timescales, remain the same. Parameter choice is extremely important in model‐based studies and can create many biases in results, however, we are fairly confident we mitigated these biases as best we could and we caution against over‐interpretation of exact timing and population sizes, and rather encourage focusing on the broader trends presented by the data.

In addition to mitigating biases, our study design provides a rich and highly informative genomic dataset capable of tackling questions regarding the impact of whaling that were unable to be addressed in previous work (see Taylor et al., 2021). We used high‐coverage, genome‐wide data, polarized SFSs, identity‐by‐descent tracts and runs of homozygosity to both describe diversity and investigate changes in effective population size, with fine scale resolution thanks to the use of dedicated methods to analyse demographic changes from the times of divergence to the near present (~4 generations ago). Whole genome datasets and the increasing availability of reference genomes are opening doors to help answer new and long‐standing questions important for conservation (Formenti et al., 2022; Theissinger et al., 2023).

When tackling real‐world conservation issues and trying to save a species from the brink of extinction, there are often obvious (while extremely difficult to achieve) goals including stopping habitat destruction, preventing anthropogenic mortalities and protecting critical habitat, among others. What is less clear, however, is when do we consider a species or population to be ‘recovered’. The identification of clear and specific goals in conservation is often a challenge. Genomic data are proving to be an invaluable tool for conservation management providing more accurate measures of genetic diversity, a better understanding of the genetics of small populations and estimates of demographic history of wildlife populations (Formenti et al., 2022; Grueber & Sunnucks, 2022; Kardos et al., 2021; Paez et al., 2022; Taylor et al., 2021; Theissinger et al., 2023). Understanding historical effective population sizes and how they have changed over different time scales can identify onset of anthropogenic impacts and can help determine what a stable effective population size for a species might be. We can use genomic data to identify long‐term small population sizes and subsequently predict the impact of inbreeding and/or genetic load on fitness and population viability (i.e. Dussex et al., 2021; Robinson et al., 2022). When comparing populations or species, such as between right whales in this study, these historical effective population sizes should not be the target for census size themselves but can help in setting different expectations of long‐term recovery for different populations or species. The vastly different effective population sizes between extant North Atlantic and Southwest Atlantic southern right whales throughout much of their history identified in our results, paired with the differences in reproductive output and inbreeding coefficients, suggest the recovery potential of these two populations may be very different and therefore using this southern right whale population as a benchmark for recovery in North Atlantic right whales is likely not appropriate.

Limiting anthropogenic mortality is essential to the conservation of North Atlantic right whales, but we can also now begin to understand how the genomic landscape will affect the subsequent population recovery. Although the combination of long‐term small *N*_*e*_, low levels of diversity and recent inbreeding put North Atlantic right whales in a situation where genetic factors are likely to be impacting individual fitness and species recovery, the situation may not be as bleak as it initially sounds. The field of conservation biology has numerous success stories of species brought to very small numbers and low levels of genetic diversity, but which were still able to recover. Some examples include the northern elephant seal (Hoelzel, 1999), the Channel Island foxes (Robinson et al., 2018), the Chatham Island black robin (von Seth et al., 2022) and the kākāpō (Dussex et al., 2021). Therefore, although quantifying the impacts of genetic characteristics (including genetic load) on individual fitness and species recovery are important next steps we are carrying out, the overall forecast for the species should still be positive, particularly given the fact that the population has increased in the past—although at a modest rate—during periods of lower mortality (e.g. Pace et al., 2017).

## AUTHOR CONTRIBUTIONS

*Project Conceptualization*: TRF & CAC. *Study Design*: TRF, CAC & MCF. *Sample Selection and Processing*: CAC & TRF. *Data Analysis*: CAC & MCF. *Manuscript Preparation*: CAC, with contributions by TRF & MCF.

## FUNDING INFORMATION

This study was supported by funding and support provided by Genome Canada and Genome Atlantic, Research Nova Scotia and an NSERC Discovery Grant awarded to TRF. CAC was supported by an NSERC‐CGS, NSERC MSFSS and an SMU VPAR International Mobility award.

## CONFLICT OF INTEREST STATEMENT

The authors declare no conflict of interest.

## BENEFIT SHARING STATEMENT

These long‐term sample collections have been made possible by numerous collaborations including participation from a number of regional organizations. Specifically, we draw attention to those individuals and organizations acknowledged in Payne (1986), and Schaeff et al. (1997) for their contributions to southern right whale sample collection and recognize the number of organizations that contribute information from their region to the North Atlantic Right Whale Consortium (https://www.narwc.org). All samples were collected with the required local permits and imported/exported with CITES permits as required. The hope is that these data improve our understanding of the biology of these species, and will be used to improve conservation actions in both locations.

Just as our study species disperse across international borders, we strive to make all aspects of our project available to all stakeholders. All code, scripts and data used in this study have been made publicly available. We particularly hope that our colleagues currently studying southern right whales in Argentina (and elsewhere in the southern hemisphere) benefit from these data and can incorporate them into their ongoing analyses.

## Supporting information


Data S1.

## Data Availability

Aligned sequence reads have been deposited to the NCBI Sequence Read Archive under accession numbers SRR22863734‐SRR22863755 (BioProject: PRJNA914998). Code used in these analyses has been made publicly available: https://github.com/carlacrossman/RightWhale_WGS
