# Supplementary material for: A comparison of genomic diversity and demographic history of the North Atlantic and Southwest Atlantic southern right whales
Source: Mol Ecol. 2023 Aug 14;33(20):e17099. doi: 10.1111/mec.17099 (PMC13084989; doi:10.1111/mec.17099)
Supplement: Supplementary file 1 — Data S1. [file MEC-33-e17099-s001.pdf]

MOLECULAR ECOLOGY

Supplemental Information for:

A comparison of genomic diversity and demographic history of the North Atlantic and Southwest Atlantic southern right whales

Carla A. Crossman, Michael C. Fontaine, Timothy R. Frasier

Table of Contents:

**Supplementary Figures:**	
**Figure S1.** Flowchart depicting variant calling pipeline	**Page 2**
**Figure S2.** Flowchart depicting variant filtering steps	**Page 3**
**Figure S3.** Flowchart depicting analyses used in this study	**Page 4**
**Figure S4.** Linkage disequilibrium decay in North Atlantic and southern right whales.	**Page 5**
**Figure S5.** Summary of ADMIXTURE from PONG for North Atlantic right whales	**Page 6**
**Figure S6.** Summary of ADMIXTURE from PONG for southern right whales	**Page 7**
**Figure S7.** Cross validation error across 10 runs of ADMIXTURE	**Page 8**
**Figure S8.** Effects of different mutation rates on results from MSMC2	**Page 9**
**Figure S9.** Effects of different r^2 max parameters and recombination rates on IBDNe	**Page 10**
**Figure S10.** Results of STAIRWAY PLOT excluding the sample from South Georgia	**Page 11**
**Figure S11.** Results of IBDNe excluding the sample from South Georgia	**Page 12**
**Supplementary Tables**	
**Table S1.** Descriptive statistics for samples used in this study	**Page 13**
**Table S2.** Descriptive statistics for reference genomes used in this study	**Page 16**
**Table S3.** F_{ROH} values calculated using different length thresholds and different methods	**Page 17**
**Table S4.** Genome-wide nucleotide diversity estimates for cetaceans used in Figure 5	**Page 18**

MOLECULAR ECOLOGY

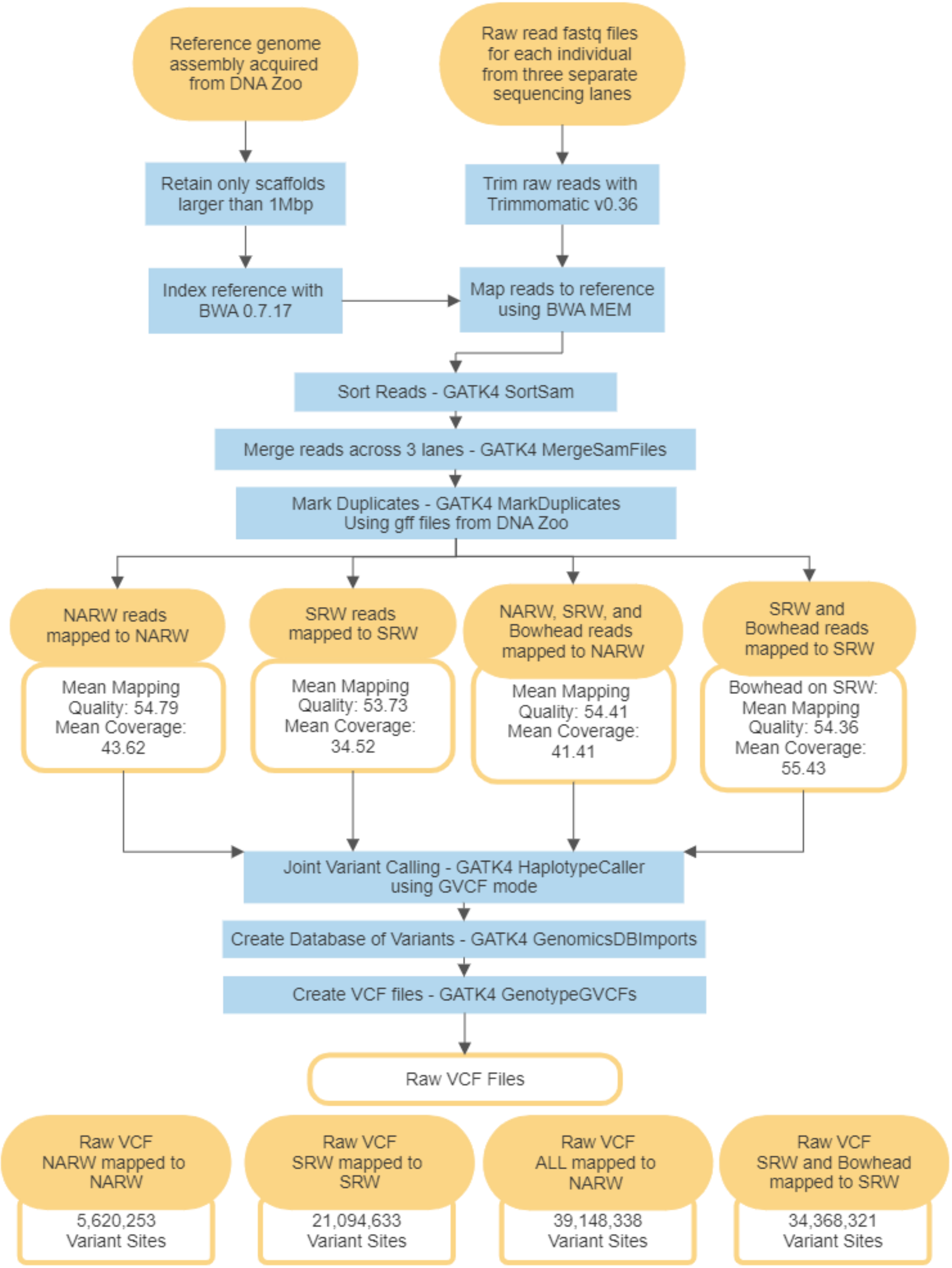

Figure S1. Variant (SNP and INDEL) calling pipeline used in this study.

MOLECULAR ECOLOGY

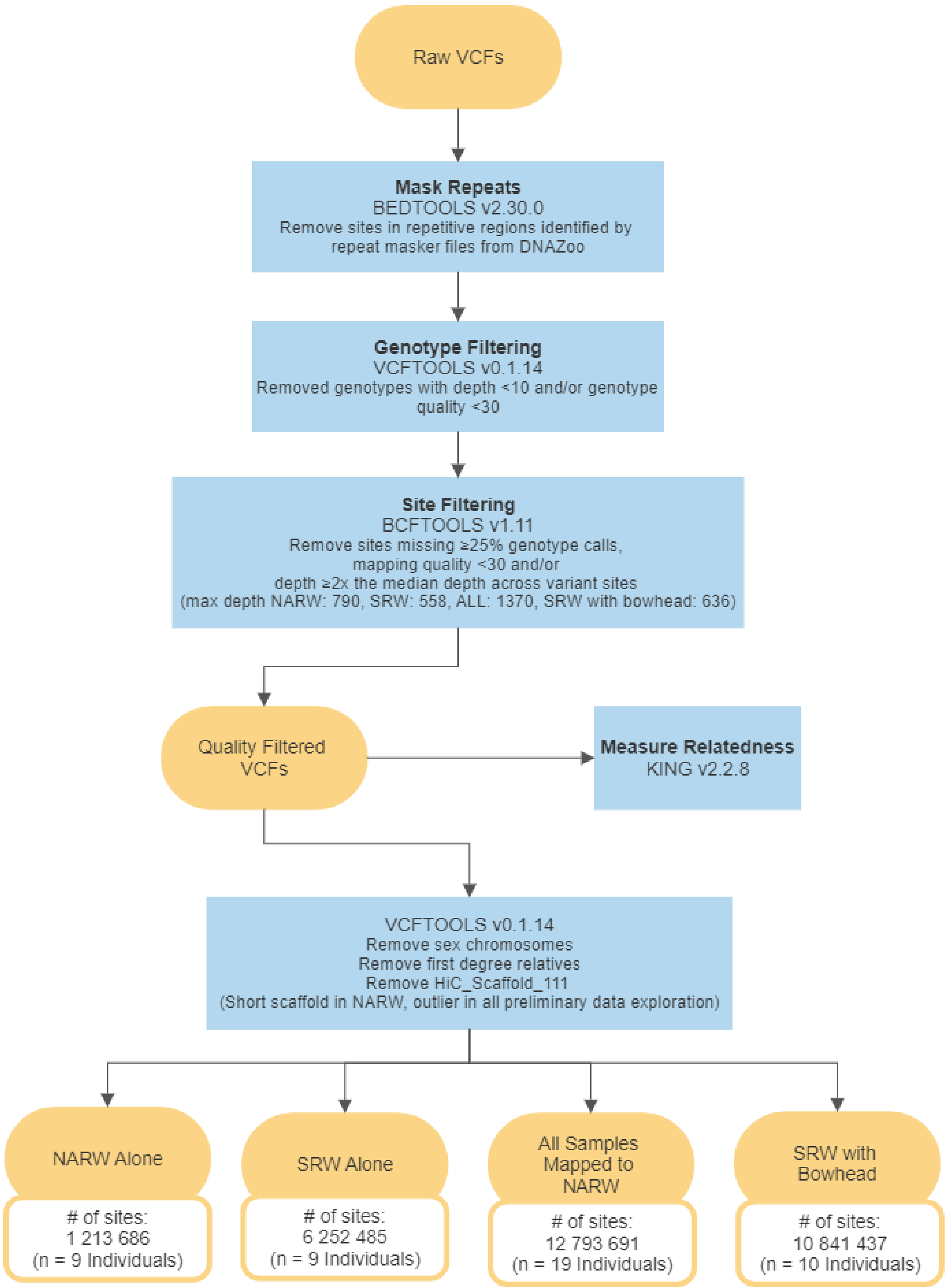

Figure S2. Variant (SNP and INDEL) filtration pipeline used in this study.

MOLECULAR ECOLOGY

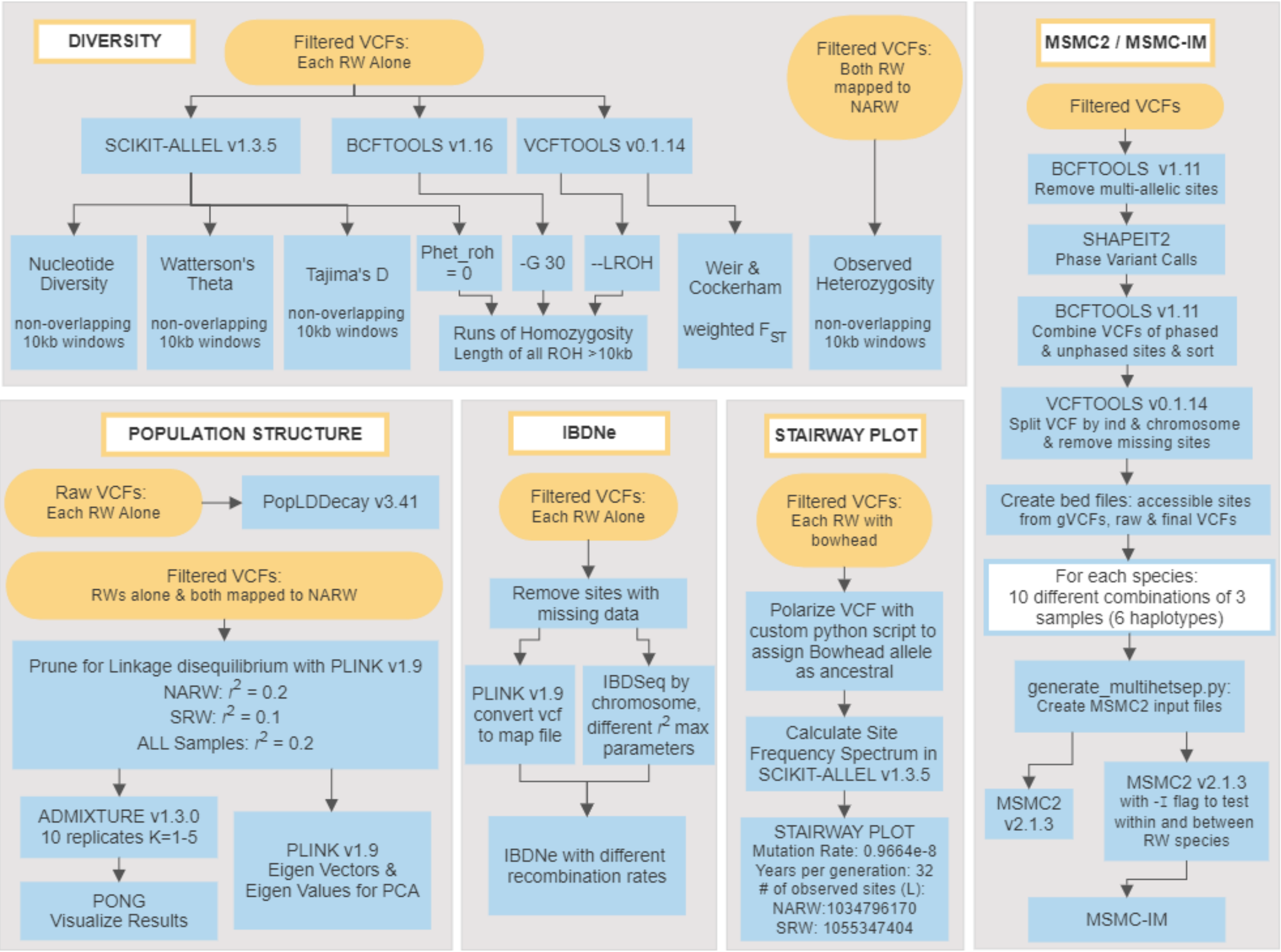

Figure S3. Schematic of tests performed in this study.

MOLECULAR ECOLOGY

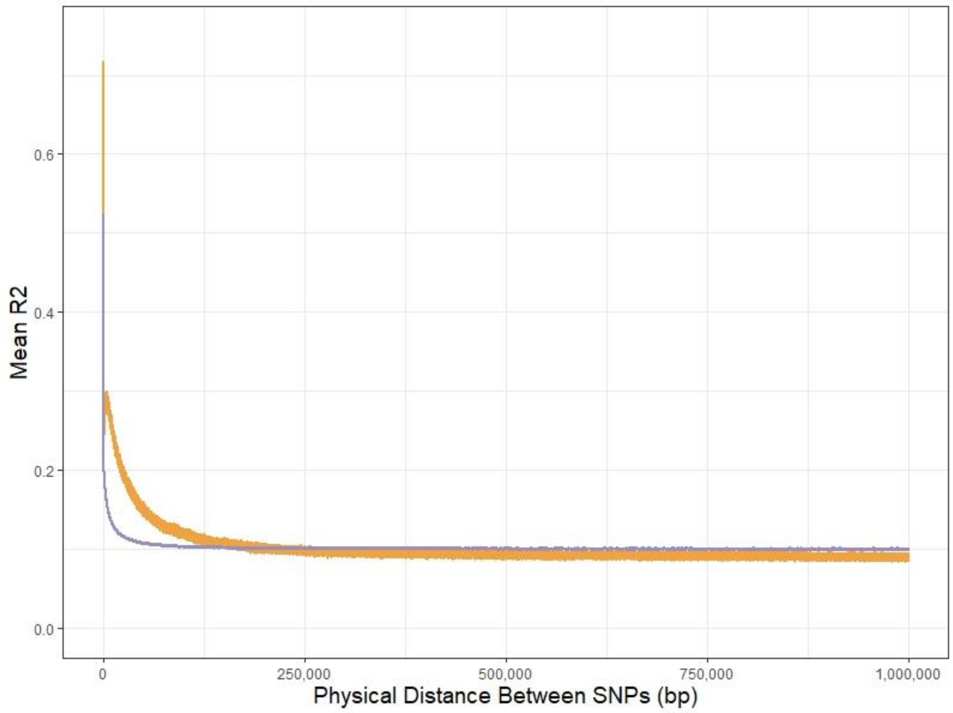

Figure S4. Decay in linkage between SNPs generated by PopLDdecay for North Atlantic (orange) and southern right whales (purple).

MOLECULAR ECOLOGY

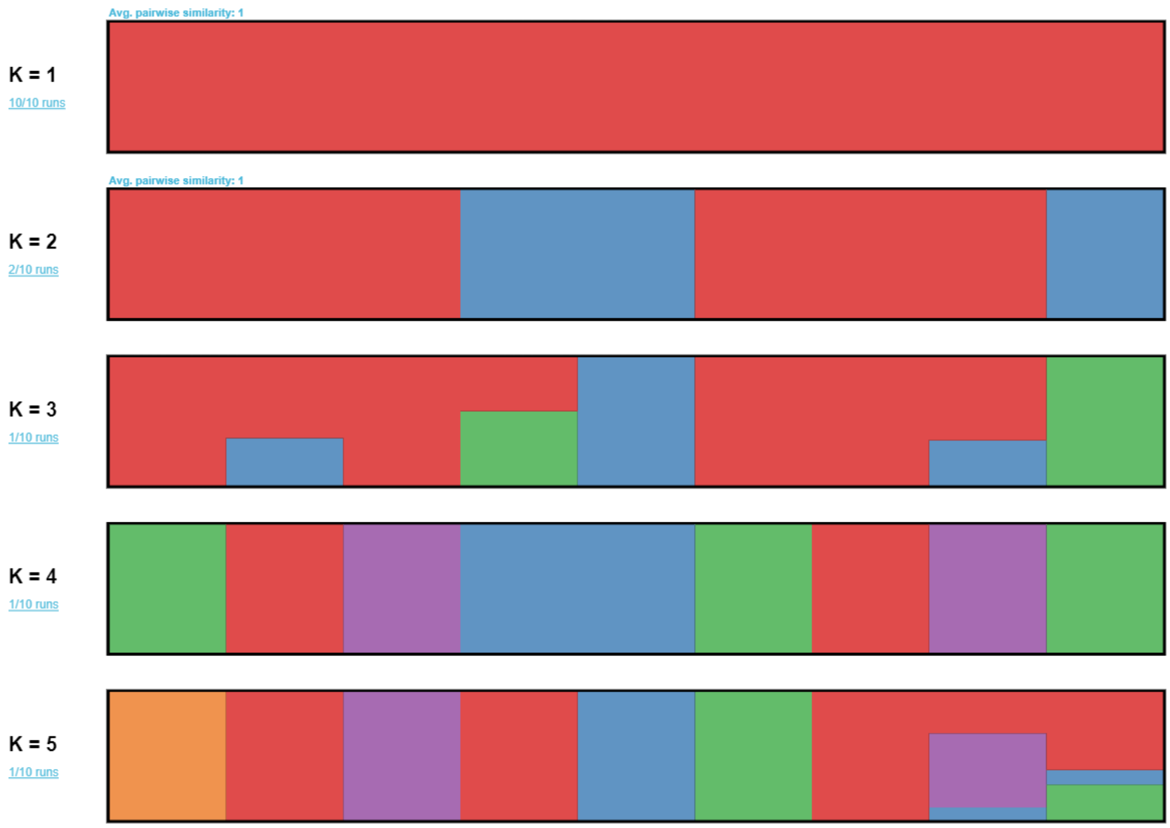

Figure S5. Visualization of 10 runs of ADMIXTURE from PONG for North Atlantic right whales. There is a lack of convergence across values of K>1.

MOLECULAR ECOLOGY

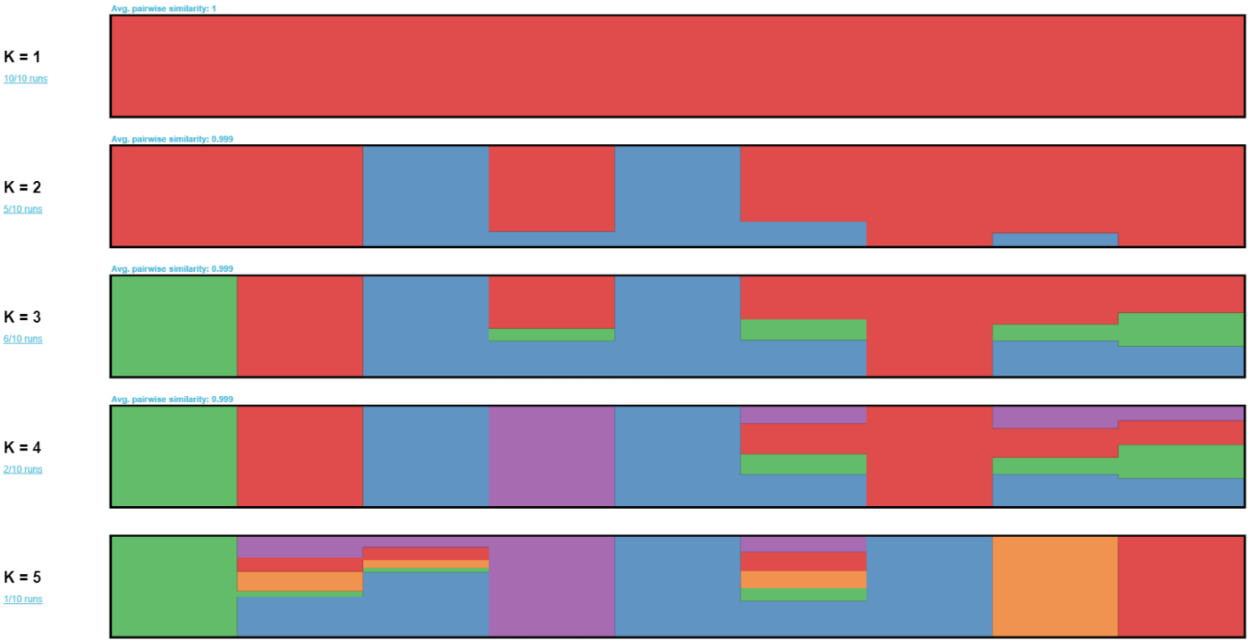

Figure S6. Visualization of 10 runs of ADMIXTURE from PONG for southern right whales. There is a lack of strong convergence across values of K>1.

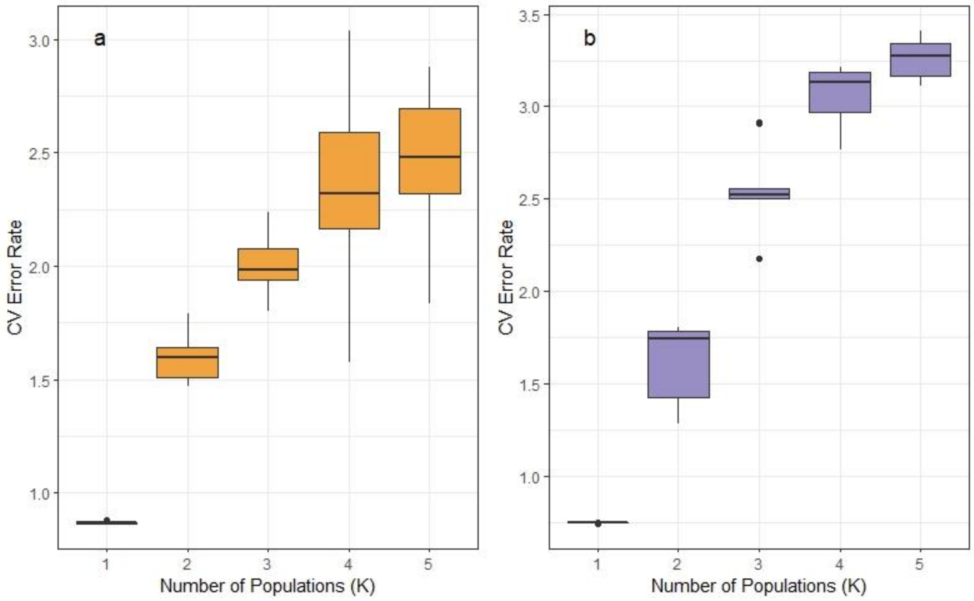

Figure S7. Cross validation error rate of 10 iterations of ADMIXTURE for different values of K for a) North Atlantic right whales and b) southern right whales.

MOLECULAR ECOLOGY

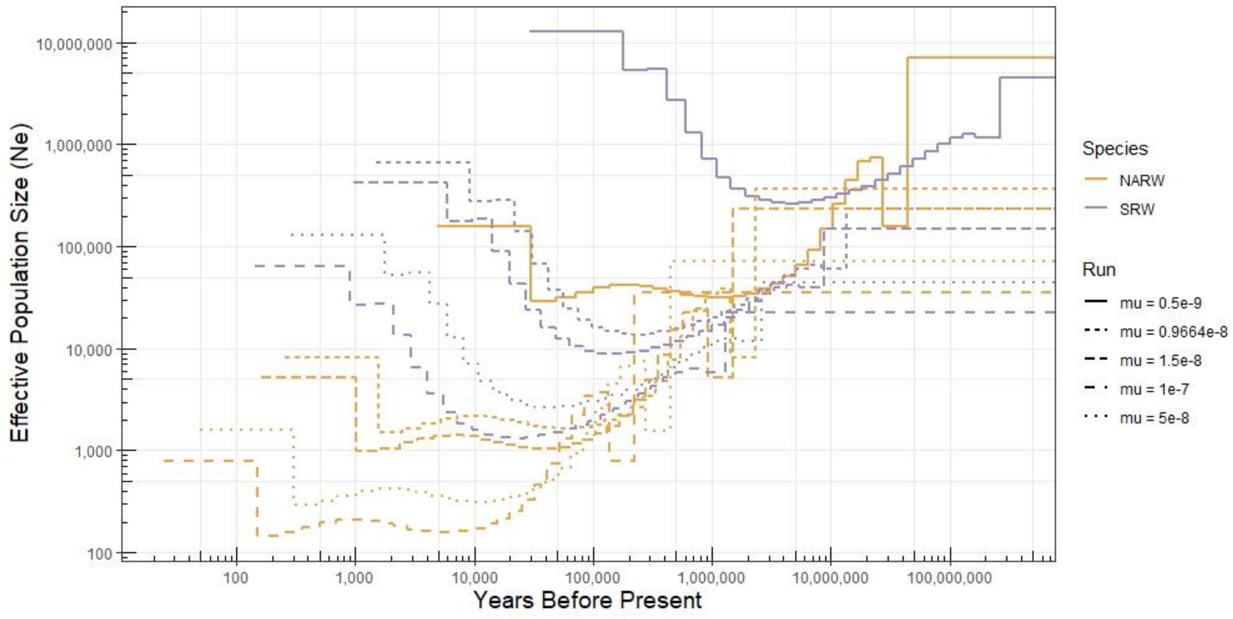

Figure S8. Effect of different mutation rates on the results of one run of MSMC on North Atlantic (orange) and southern right whales (purple). The timing of events and the effective population sizes are rescaled by the choice of mutation rate, but the broad pattern of changes in population size over time remains the same. Based on previous estimates for mysticetes (Dornburg *et al.* 2012), we used a mutation rate of 0.9664e-8 mutations/site/generation in this study.

MOLECULAR ECOLOGY

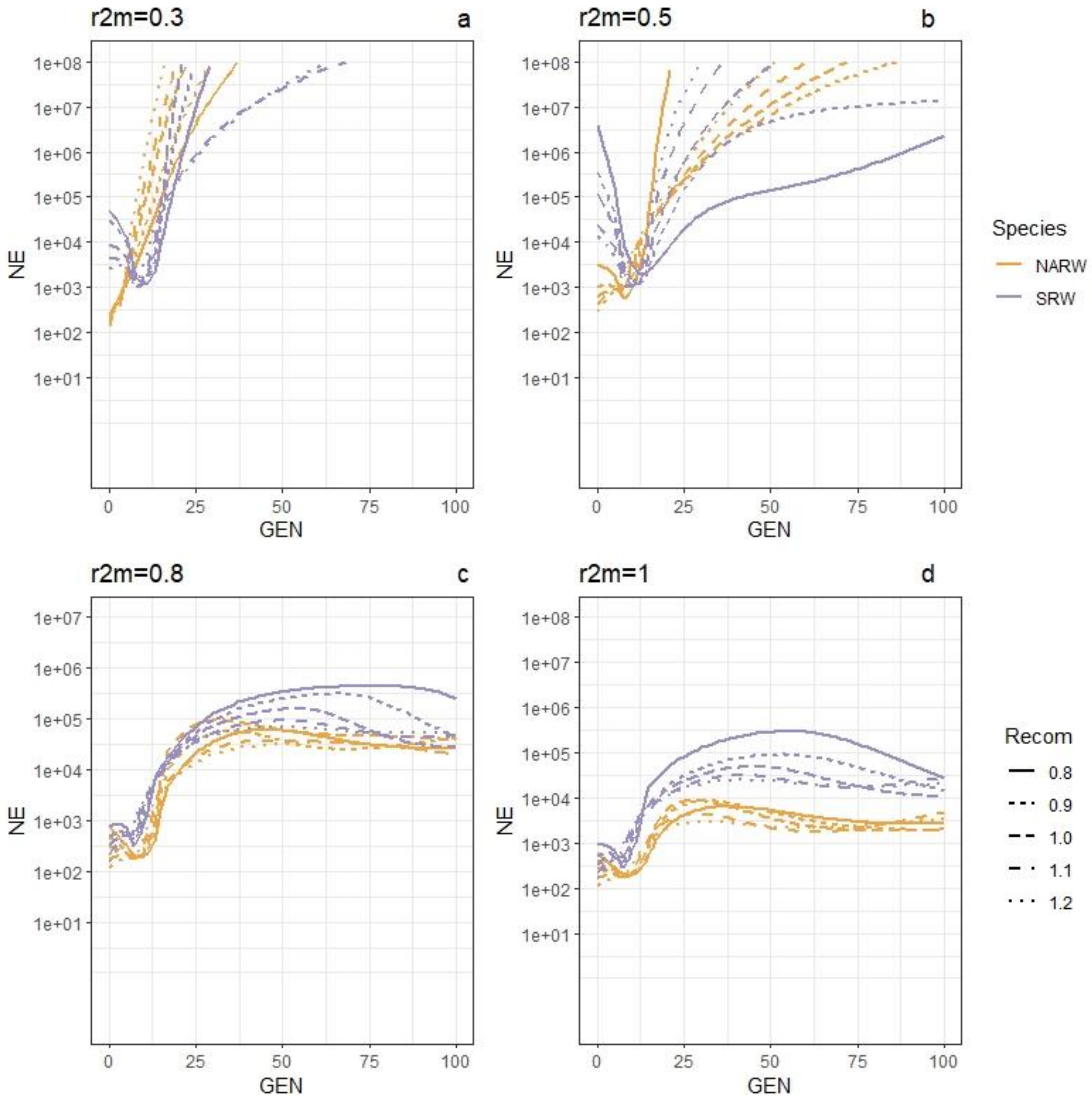

Figure S9. Effect of recombination rate and r²max parameter choice on estimates of effective population size (N_e) of North Atlantic (orange) and southern (purple) right whales generated by IBDNe.

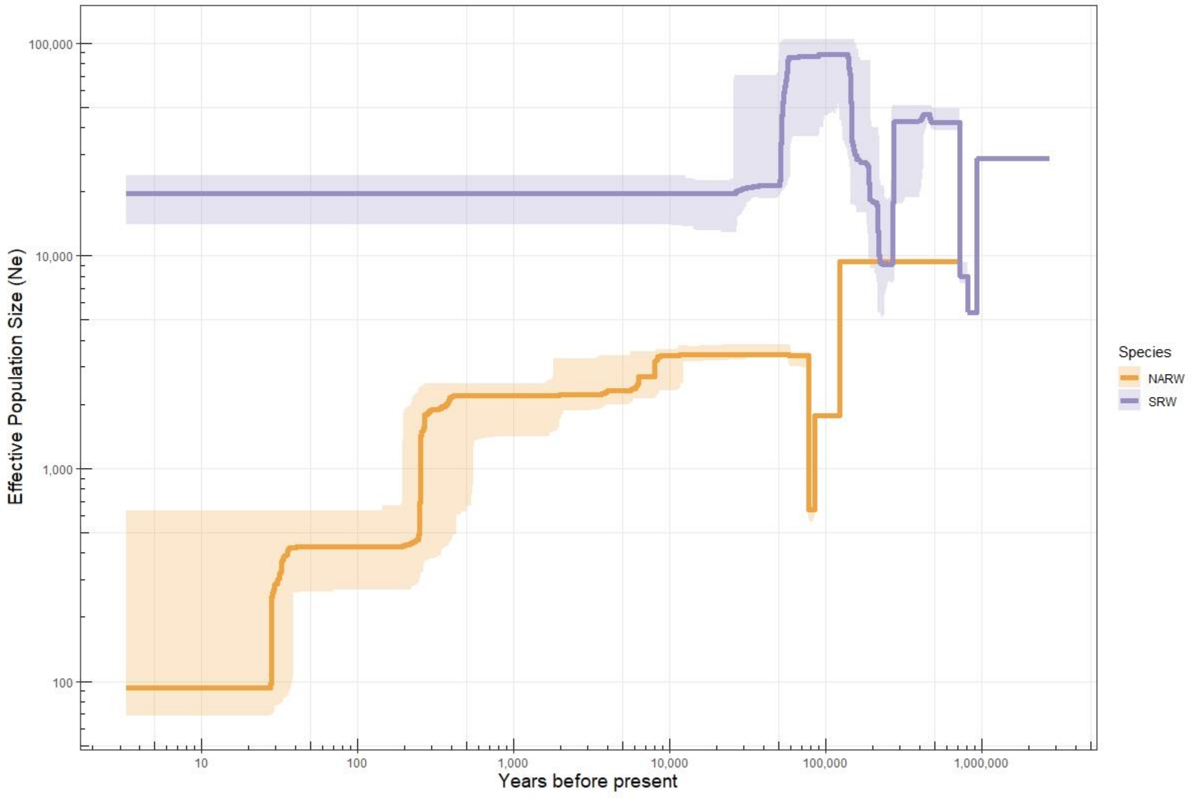

Figure S10. Estimates of effective population size of North Atlantic (orange) and southern right whales (purple) through time based on the site frequency spectrum in STAIRWAY PLOT2 excluding one southern right whale sample (Eau283) collected from South Georgia to confirm our main findings were not influenced by subtle population structure. The shaded regions represent the 2.5%-97.5% confidence limits for 200 estimates.

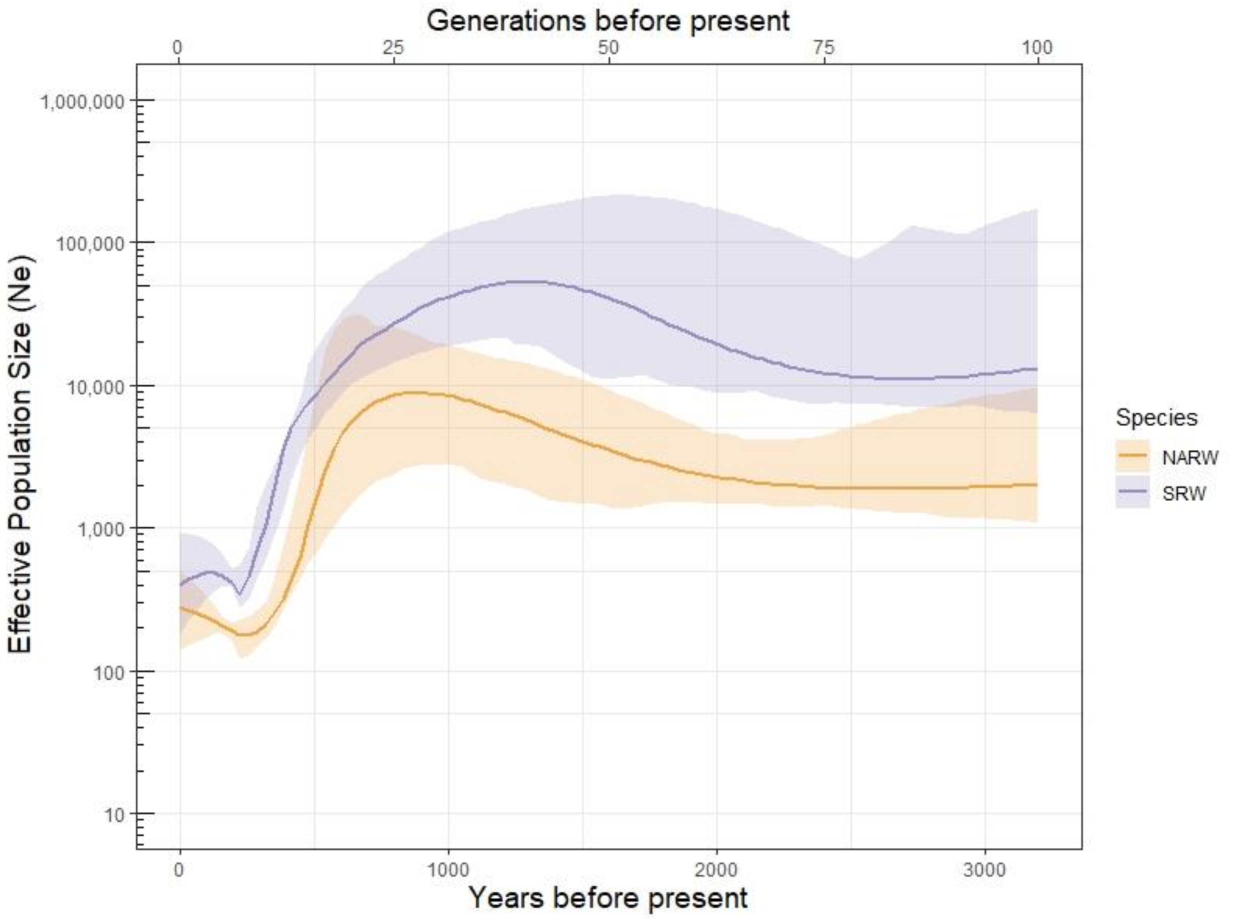

Figure S11. Effective population size in recent history as estimated by IBDNe for North Atlantic (orange) and southern right whale (purple) excluding one southern right whale sample (Eau283) collected from South Georgia to confirm our results were not influenced by subtle population structure. Key parameters in this additional test were the same as presented in the main text: a constant recombination rate of 1.0 cm/Mb, an r2max = 1.0 and a generation time of 32 years. 95% confidence intervals are depicted by the shaded areas.

MOLECULAR ECOLOGY

Table S1. Descriptive statistics for samples used in this study. The latter statistics pertain to results from mapping to their respective species' reference assembly.

Sample ID	Ind ID	Species	Sex	Sample Collection Location	Sample Collection Date	NCBI Accession ID	Total number of raw reads	Sequencing Depth (mean±SD)	% of genome covered by ≥10X	Variable sites with genotype calls passing filters	Ind hetero-zygosity (F)
**Egl 00252-1**	NEA 1706	NARW	F	Bay of Fundy 44.68833, -66.46167	Aug 4, 1997	SRR22863755	357,409,922	41.45 ± 2102.56	99.02	1,198,058	-0.08305
**Egl 254-1**	NEA 1209	NARW	F	Bay of Fundy 44.585, -66.63167	Aug 18, 1997	SRR22863754	461,289,854	53.92 ± 2479.9	99.06	1,200,876	-0.09288
**Egl 308-1a**	NEA 1968	NARW	F	Bay of Fundy 44.64667, -66.37333	Sept 21, 1998	SRR22863743	412,868,996	47.56 ± 2223.27	97.89	1,195,032	-0.10973
**Egl 013-3qa**	NEA 1027	NARW	F	Cape Cod Bay 41.88333, -70.11833	Mar 19, 1997	SRR22863740	157,879,013	18.16 ± 858.76	81.18	942,617	-0.13296
**Egl 183-1**	NEA 2040	NARW	F	Bay of Fundy 44.55, -66.41333	Aug 27, 1995	SRR22863739	738,310,854	85.59 ± 5541.17	99.10	1,202,668	-0.05865
**Egl 276-1†**	NEA 1934	NARW	F	Bay of Fundy, 44.605, -66.515	Sept 11, 1997	SRR22863738	233,564,107	26.92 ± 1136.15	98.31	–	–
**Egl 312-1a†**	NEA 1240	NARW	F	Bay of Fundy 44.68167, -66.50833	Aug 17, 1995	SRR22863737	443,996,567	50.61 ± 3061.98	97.98	–	–
**Egl 336_1b**	NEA 1315	NARW	F	Florida, 30.635, -81.22667	Jan 12, 1998	SRR22863736	378,965,079	43.01 ± 2696.75	83.22	955,357	-0.10277
**SID 179132**	NEA 1204	NARW	F	Florida 30.45833, -81.14167	Mar 7, 2009	SRR22863735	453,242,967	52.66 ± 2371.98	99.07	1,200,280	-0.08794

MOLECULAR ECOLOGY

Sample ID	Ind ID	Species	Sex	Sample Collection Location	Sample Collection Date	NCBI Accession ID	Total number of raw reads	Sequencing Depth (mean±SD)	% of genome covered by ≥10X	Variable sites with genotype calls passing filters	Ind hetero-zygosity (F)
**SID 181803[†]**	NEA 1334	NARW	F	Florida 30.27573, -81.30452	Feb 21, 2013	SRR22863734	245,549,525	28.61 ± 1541.08	95.59	–	–
**Egl 140-1**	NEA 1131	NARW	M	Roseway Basin, 42.89333, -65.36167	Sept 10, 1991	SRR22863753	297,155,891	34.51 ± 1763.5	98.46	1,191,098	-0.10677
**Egl 272-1**	NEA 1037	NARW	M	Bay of Fundy, 44.61, -66.43	Aug 18, 1997	SRR22863752	348,623,351	40.49 ± 1953.07	98.90	1,197,656	-0.10701
**Eau 7**	N/A	SRW	M	Argentina	1988	SRR22863751	184,153,280	19.66 ± 1357.24	74.48	5,799,668	-0.00221
**Eau 9c**	N/A	SRW	F	Peninsula Valdes	1989	SRR22863750	226,812,439	24.73 ± 1532.19	91.54	6,013,151	-0.00217
**Eau 10b**	N/A	SRW	M	Peninsula Valdes	1989	SRR22863749	518,434,524	54.39 ± 2983.71	93.89	6,048,321	-0.61309
**Eau 017**	N/A	SRW	F	Peninsula Valdes	1989	SRR22863748	394,616,387	42.48 ± 2617.96	93.14	6,239,654	0.01471
**Eau 018**	N/A	SRW	M	Peninsula Valdes	1989	SRR22863747	303,091,742	32.15 ± 2518.82	80.42	5,997,087	-0.00114
**Eau 019[†]**	N/A	SRW	F	Peninsula Valdes	1989	SRR22863746	496,312,699	50.16 ± 4145.34	79.43	–	–
**Eau 023**	N/A	SRW	M	Peninsula Valdes	1989	SRR22863745	345,538,989	36.6 ± 2786.91	82.92	6,175,009	0.01386
**Eau 029**	N/A	SRW	F	Peninsula Valdes	1989	SRR22863744	232,417,002	24.81 ± 1896.21	76.12	5,955,205	0.00860

MOLECULAR ECOLOGY

Sample ID	Ind ID	Species	Sex	Sample Collection Location	Sample Collection Date	NCBI Accession ID	Total number of raw reads	Sequencing Depth (mean±SD)	% of genome covered by ≥10X	Variable sites with genotype calls passing filters	Ind hetero-zygosity (F)
**Eau 034A**	N/A	SRW	F	Peninsula Valdes	1989	SRR22863742	419,385,099	44.58 ± 4889.47	88.64	6,206,385	0.02182
**Eau 283**	N/A	SRW	M	South Georgia	1989	SRR22863741	148,959,155	15.6 ± 883.38	75.28	4,845,130	0.02488

† These samples were removed from analyses due to high relatedness with another sample (equivalent to first-order relatives or closer).

MOLECULAR ECOLOGY

Table S2. Descriptive statistics of the reference genomes used in this study

	North Atlantic right whale	Southern right whale
**Total Scaffold Length**	2,369,417,546 bp	2,316,908,615 bp
**Number of Scaffolds**	172,124	3,234
**Scaffold N50**	101,413,572 bp	112,042,483 bp
**Number of Scaffolds >1Mb**	23	21
**Size of Reference Genome Scaffolds >1Mb**	2,170,759,585 bp	2,296,311,778 bp

MOLECULAR ECOLOGY

Table S3. *F_{ROH}* estimates generated with different methods and different thresholds for identifying ROHs.

		BCFTOOLS			SCIKIT-ALLEL			VCFTOOLS		
**SAMPLE**	Species	FROH 100Kb	FROH 300Kb	FROH 1Mb	FROH 100Kb	FROH 300Kb	FROH 1Mb	FROH 100Kb	FROH 300Kb	FROH 1Mb
**EGL00252-1**	NARW	0.095	0.058	0.021	0.136	0.038	0.007	0.069	0.034	0.009
**Egl013-3qa**	NARW	0.064	0.036	0.009	0.166	0.031	0.003	0.054	0.026	0.003
**Egl140-1**	NARW	0.080	0.042	0.010	0.121	0.024	0.001	0.054	0.020	0.002
**Egl183-1**	NARW	0.119	0.081	0.029	0.165	0.057	0.008	0.089	0.053	0.008
**Egl254-1**	NARW	0.093	0.054	0.016	0.132	0.034	0.004	0.061	0.031	0.007
**Egl272-1**	NARW	0.071	0.034	0.001	0.113	0.014	0.000	0.047	0.016	0.000
**Egl308-1a**	NARW	0.091	0.057	0.015	0.135	0.039	0.003	0.070	0.038	0.006
**Egl336_1b**	NARW	0.071	0.041	0.015	0.170	0.033	0.005	0.054	0.024	0.004
**SID179132**	NARW	0.088	0.051	0.015	0.131	0.034	0.006	0.063	0.029	0.004
	**MEAN NARW**	0.086	0.050	0.014	0.141	0.034	0.004	0.062	0.030	0.005
	**SD NARW**	0.017	0.015	0.008	0.021	0.012	0.003	0.012	0.011	0.003
**Eau017**	SRW	0.020	0.006	0.001	0.009	0.002	0.000	0.008	0.002	0.000
**Eau018**	SRW	0.019	0.003	0.001	0.010	0.001	0.000	0.009	0.000	0.000
**Eau023**	SRW	0.022	0.006	0.004	0.012	0.003	0.000	0.010	0.003	0.001
**Eau029**	SRW	0.019	0.005	0.000	0.010	0.001	0.000	0.008	0.001	0.000
**Eau034A**	SRW	0.025	0.011	0.003	0.015	0.006	0.002	0.014	0.007	0.002
**Eau10b**	SRW	0.000	0.000	0.000	0.000	0.000	0.000	0.000	0.000	0.000
**Eau283**	SRW	0.050	0.037	0.024	0.043	0.025	0.005	0.024	0.014	0.005
**Eau7**	SRW	0.020	0.005	0.000	0.011	0.001	0.000	0.008	0.001	0.000
**Eau9c**	SRW	0.027	0.013	0.007	0.014	0.003	0.000	0.009	0.003	0.001
	**MEAN SRW**	0.023	0.010	0.004	0.014	0.005	0.001	0.010	0.004	0.001
	**SD SRW**	0.013	0.011	0.008	0.012	0.008	0.002	0.006	0.004	0.002

MOLECULAR ECOLOGY

Table S4. Genome-wide nucleotide diversity estimates for cetaceans used in Figure 5. Data were compiled in Robinson et al. (2016) and Morin et al. (2021), except those from right whales generated in this study.

Species	Nucleotide diversity (π)	Original Source(s)
**Vaquita (*Phocoena sinus*)**	0.00010	Morin et al. 2021
**Baiji (*Lipotes vexillifer*)**	0.00012	Zhou et al. 2013.
**Narwhal (*Monodon monoceros*)**	0.00014	Westbury et al. 2019.
**North Atlantic right whale (*Eubalaena glacialis*)**	0.00019	This study
**Killer whale (*Orcinus orca*)**	0.00021	Westbury et al. 2018.
**Beluga whale (*Delphinapterus leucas*)**	0.00029	Westbury et al. 2019.
**Minke whale (*Balaenoptera acutorostrata*)**	0.00061	Yim et al. 2014.
**Southern right whale (*Eubalaena australis*)**	0.00080	This study
**Finless porpoise (*Neophocaena phocaenoides*)**	0.00086	Yim et al. 2014.
**Indo-Pacific finless porpoise (*Neophocaena phocaenoides*)**	0.00093	Morin et al. 2021. Zhou et al. 2018.
**Yangtze finless porpoise (*Neophocaena asiaeorientalis asiaeorientalis*)**	0.00105	Morin et al. 2021. Zhou et al. 2018.
**Narrow-ridged finless porpoise (*Neophocaena asiaeorientalis*)**	0.00134	Morin et al. 2021. Zhou et al. 2018.
**Bottlenose dolphin (*Tursiops truncatus*)**	0.00142	Yim et al. 2014.
**Fin whale (*Balaenoptera physalus*)**	0.00151	Yim et al. 2014.
**Blue whale (*Balaenoptera musculus*)**	0.00210	Morin et al. 2021.
**Sperm whale (*Physeter macrocephalus*)**	0.00228	Morin et al. 2021.; Fan et al. 2019.

MOLECULAR ECOLOGY

Table S4 References

Fan G, Zhang Y, Liu X, Wang J, Sun Z, Sun S, *et al.* (2019). The first chromosome-level genome for a marine mammal as a resource to study ecology and evolution. *Mol Ecol Resour* **19**: 944–956.

Morin PA, Archer FI, Avila CD, Balacco JR, Bukhman Y V., Chow W, *et al.* (2021). Reference genome and demographic history of the most endangered marine mammal, the vaquita. *Mol Ecol Resour* **21**: 1008–1020.

Robinson JA, Ortega-Del Vecchyo D, Fan Z, Kim BY, Vonholdt BM, Marsden CD, *et al.* (2016). Genomic Flatlining in the Endangered Island Fox. *Curr Biol* **26**: 1183–1189.

Westbury M V., Hartmann S, Barlow A, Wiesel I, Leo V, Welch R, *et al.* (2018). Extended and continuous decline in effective population size results in low genomic diversity in the world's rarest hyena species, the brown Hyena. *Mol Biol Evol* **35**: 1225–1237.

Westbury M V., Petersen B, Garde E, Heide-Jørgensen MP, Lorenzen ED (2019). Narwhal Genome Reveals Long-Term Low Genetic Diversity despite Current Large Abundance Size. *iScience* **15**: 592–599.

Yim HS, Cho YS, Guang X, Kang SG, Jeong JY, Cha SS, *et al.* (2014). Minke whale genome and aquatic adaptation in cetaceans. *Nat Genet* **46**: 88–92.

Zhou X, Sun F, Xu S, Fan G, Zhu K, Liu X, *et al.* (2013). Baiji genomes reveal low genetic variability and new insights into secondary aquatic adaptations. *Nat Commun* **4**: 2708.

Zhou X, Guang X, Sun D, Xu S, Li M, Seim I, *et al.* (2018). Population genomics of finless porpoises reveal an incipient cetacean species adapted to freshwater. *Nat Commun* **9**: 1276
